# The influence of collaborative music creation supported by generative artificial intelligence on students’ creativity

**DOI:** 10.3389/fpsyg.2025.1709513

**Published:** 2026-01-06

**Authors:** Ziqiu Zhuang, Xue Li

**Affiliations:** Graduate School, Department of Education, Gachon University, Seongnam, Republic of Korea

**Keywords:** collaborative learning, creativity, generative AI, learning achievement, music education

## Abstract

**Introduction:**

The integration of generative artificial intelligence (GAI) into education is advancing rapidly, and its potential in creative learning contexts is gaining increasing attention.However, in the domain of music education, the mechanisms through which GAI exerts its influence remain underexplored.

**Methods:**

This study investigates the effects of GAI-supported collaborative music creation on college students’ creative interest, creative self-efficacy, self-regulated learning ability, and perceived creative competence. Employing a mixed-method approach that combines structural equation modeling (SEM) with experimental design, the study analyzes data from a sample of 405 university students in China.

**Results:**

The results reveal that GAI support significantly enhances creative interest (*β* = 0.616), creative self-efficacy (*β* = 0.557), self-regulated learning (*β* = 0.473), and perceived creative competence (*β* = 0.357). Furthermore, creative interest, creative self-efficacy, and self-regulated learning are all significant predictors of perceived creative competence. A comparison between the experimental and control groups further confirms that GAI-supported collaboration significantly improves students’ creative development.

**Discussion:**

These findings offer empirical support for a theoretical model of AI-enhanced creativity and provide valuable insights for the design and implementation of intelligent music education environments.

## 1 Introduction

Creativity is a fundamental component of core literacy and has long been regarded as essential to human development (Beaty and Kenett, 2023). With the rapid advancement of artificial intelligence technologies, the importance of creativity has become even more pronounced (Ivcevic and Grandinetti, 2024). Rather than being an isolated trait, creativity is deeply intertwined with an individual’s knowledge, skills, and cognitive abilities (Ingold and Hallam, 2021). In the context of music education, musical creativity constitutes a central aspect of students’ musical literacy. It not only reflects their unique understanding and expression of music but also serves as a driving force behind the ongoing evolution of musical art (Creech et al., 2023).

Music creation plays a vital role in cultivating students’ creativity within educational settings (Sangiorgio, 2022). Through active engagement in creative musical tasks, students are able to explore, experiment, and innovate within authentic musical contexts. These activities allow for the integration of theoretical knowledge with hands-on practice, thereby unlocking musical potential and fostering higher levels of musical creativity. Meanwhile, artificial intelligence technologies have increasingly been integrated into classroom practices to improve instructional efficiency. The emergence of Generative Artificial Intelligence (GAI) has sparked widespread debate regarding its influence on human creativity. By significantly expanding human cognitive and productive capacities, GAI has facilitated a shift in educational goals toward the development of higher-order thinking skills. A deeper exploration of GAI in music education is therefore essential to optimizing human-AI collaborative creation models and maximizing its pedagogical value (Sengar et al., 2024).

With the growing integration of GAI into educational contexts, an increasing number of scholars have begun to explore its potential in music creation and music education. For instance, Mihăescu and Mihăescu (2023) provided a comprehensive overview of various types of generative algorithms and music composition-specific software, illustrating the feasibility of integrating GAI into music creation workflows. In the realm of human–AI collaboration, Suh et al. (2021) investigated the role of GAI in facilitating co-creative processes and suggested that the presence of AI may alter the social dynamics of human collaboration. Similarly, Louie et al. (2020) examined how GAI can assist novice musicians in collaborative composition tasks, particularly through innovative modes of interactive control, and offered valuable insights into the design of effective co-creative experiences. GAI is not only reshaping the process of creation but also influencing the historical and stylistic dimensions of musical works. Wissner (2024) explored how GAI can be used in music classrooms to generate compositions in diverse styles, thereby deepening students’ understanding of music history while fostering their creative abilities.

Overall, the application of GAI in music creation and education is steadily expanding. Scholars have conducted extensive investigations across multiple dimensions, including technology, collaboration, pedagogy, and creative experience. However, despite these advances, there remains a lack of practical implementation of GAI-supported models in actual music creation instruction. To address this gap, the present study integrates GAI into music composition teaching to foster students’ creativity.

## 2 Theories and hypotheses

This study focuses on creative interest, creative self-efficacy, and self-regulated learning. Creative interest is closely associated with sustained engagement, the exploration of multiple solutions, and the accumulation of knowledge, which are key elements for creative development (Pagel and Kwiatkowski, 2003). Creative self-efficacy reflects learners’ beliefs in their ability to generate novel and useful ideas and has been shown to predict perceived competence and intrinsic motivation across various learning contexts (Haase et al., 2018). Self-regulated learning enables learners to effectively plan, monitor, and adjust their learning processes, thereby enhancing their ability to translate motivational states into actual creative outcomes (Rubenstein et al., 2018). Together, these variables provide a comprehensive framework for understanding how learners’ affective-motivational and cognitive regulation processes influence their perceived creative competence.

### 2.1 Generative AI support and creative interest

The emergence of interest is often triggered by immediate and engaging features in the environment, such as the novelty of the task, its personal relevance, and the presence of social support (Renninger et al., 2019). GAI, with its dynamic, interactive, and highly personalized features, serves as a powerful catalyst for stimulating learners’ interest in creative tasks (Aad and Hardey, 2025).

The novelty of GAI lies in its capacity to continuously produce unique and unexpected musical elements—such as melodies, harmonic progressions, and lyrical ideas (Han et al., 2024). This generative ability can effectively capture learners’ attention, spark curiosity, and stimulate a desire for exploration. Notably, GAI offers immediate, non-judgmental feedback and suggestions, serving a scaffolding function throughout co-creative iterations (Chen et al., 2024). By supporting learners in real time, GAI helps foster key components of situational interest, allowing students to engage more deeply in collaborative creative activities. Previous studies have demonstrated that GAI can significantly enhance students’ interest in engaging with complex tasks (Hmoud et al., 2024). Learning environments that leverage AI-generated content enrich learners’ sense of novelty and autonomy, thereby sustaining their interest in creative and problem-solving activities over time (Guo et al., 2024). Furthermore, GAI-supported environments foster curiosity and deeper engagement through continuous creative output and personalized guidance (Yao et al., 2025; Saeliang and Chatwattana, 2025). Overall, the evidence indicates that GAI’s adaptive and innovative features support the enhancement of learners’ creative interest across different educational contexts.

*Hypothesis 1 (H1)*: Generative AI support has a significantly positive effect on students’ creative interest.

*Hypothesis 2 (H2)*: Compared with the control group, students using GAI showed a higher level of creative interest.

Any alternative text (alt text) provided alongside figures in this article has been generated by Frontiers with the support of artificial intelligence and reasonable efforts have been made to ensure accuracy, including review by the authors wherever possible. If you identify any issues, please contact us.

### 2.2 Generative AI support and creative self-efficacy

An individual’s self-efficacy is primarily developed through three core mechanisms: mastery experiences, vicarious learning, and verbal persuasion (Bandura, 1997). When learners collaborate with GAI to complete creative tasks—such as music or text composition—these mechanisms can be further activated and reinforced.

The guided co-creation functionality of GAI breaks down complex creative tasks into manageable, actionable steps, enabling novice learners to gradually engage with and contribute to the completion of a full creative product (Wang et al., 2024). This structured scaffolding fosters a sense of competence and progression, which is essential for the development of mastery experiences.

The GAI has been shown to enhance learners’ self-efficacy across multiple domains. For example, the use of GAI-based tools has been demonstrated to improve students’ programming self-efficacy by facilitating iterative creative generation and providing immediate, supportive feedback (Yilmaz and Yilmaz, 2023). In higher education, research indicates that GAI platforms can enhance undergraduate students’ writing self-efficacy and narrative intelligence by offering continuous cognitive assistance and reducing the perceived difficulty of creative tasks (Berkovich and Eyal, 2025). Furthermore, GAI has been characterized as a tool that supports lifelong learning self-efficacy, as frequent and effective engagement with GAI is associated with improvements in content creation (Pellas, 2023). Collectively, these findings suggest that GAI plays a pivotal role in enhancing learners’ self-efficacy by strengthening mastery experiences, facilitating observational learning, and providing ongoing encouragement throughout the creative process.

*Hypothesis 3 (H3)*: Generative AI support has a significantly positive effect on students’ creative self-efficacy.

*Hypothesis 4 (H4)*: Compared with the control group, students who used GAI had a higher level of self-efficacy in creativity.

### 2.3 Generative AI support and perceived creative competence

The core advantage of GAI lies in its ability to provide immediate and targeted assistance based on students’ individual needs, thereby facilitating their more effective acquisition of knowledge and stimulating their creative potential (Lang et al., 2025). Traditional teaching methods often limit students’ thinking patterns, while GAI generates diverse creative content to provide students with inspiration and ideas, helping them break through mental constraints and ignite their enthusiasm for independent creation. For example, in the writing process, generative AI can assist students in structuring their articles and polishing their language expressions, enabling them to have more confidence in creative writing (Yang, 2025). Additionally, in the field of painting, GAI also supports collaborative creation with machines, enhancing artistic expression (Bennett et al., 2024). Secondly, GAI plays a positive role in enhancing students’ cognitive understanding and knowledge construction (An et al., 2025). By analyzing students’ learning situations, generative AI can tailor teaching content to help students better understand complex knowledge points and concepts, promoting internalization and transfer of knowledge.

The GAI can effectively enhance individuals’ perceived creative competence by influencing both psychological and cognitive processes. For instance, among design students, GAI has been found to strengthen creative cognition by reducing anxiety and enhancing self-efficacy, thereby fostering greater awareness of personal creativity (Hwang and Wu, 2025). Additionally, research on interactions with GAI indicates that engaging in dialog with GAI can improve students’ perceived competence in creative problem-solving, as learners receive real-time feedback, gain practical strategies, and broaden their creative thinking (Song et al., 2025). GAI further stimulates creative performance by providing diverse conceptual associations and expanding learners’ cognitive search space (Habib et al., 2024). In collaborative learning contexts, GAI has been shown to enhance team creativity and performance in digital story creation (Wei et al., 2025). Within design education, the integration of GAI tools not only supports visual ideation but also reinforces learners’ confidence in their creative skills through continuous inspiration, feedback, and iterative design support (Lee, 2025). These studies highlight that GAI systematically enhances perceived creative competence by strengthening creative cognition, reducing barriers to innovation, and promoting sustained creative engagement.

*Hypothesis 5 (H5)*: Generative AI support has a significantly positive effect on students’ Perceived Creative Competence.

*Hypothesis 6 (H6)*: Compared with the control group, students who used GAI had a higher perceived creative competence.

### 2.4 Creative interest and perceived creative competence

Creative interest plays a central role in motivating individuals to engage in creative tasks and achieve deep involvement (Eisenberger and Armeli, 1997). When learners develop intrinsic interest in a task, their behavioral patterns often shift accordingly (Wiernik et al., 2016). They become more inclined to actively explore multiple solutions, persist through complex challenges, and tolerate uncertainty throughout the creative process. This interest-driven immersion allows learners to accumulate a rich body of tacit knowledge through experiential engagement. Over time, such sustained participation not only enhances their creative competence but also contributes to a gradual reconstruction of how they perceive their own creative potential.

The GAI amplifies this developmental process in two critical ways. First, its dynamic and interactive nature continuously stimulates and sustains learners’ exploratory motivation, helping to prevent the decline of interest that often results from technical difficulties. Second, by lowering technological barriers and offering real-time, formative feedback, GAI ensures that each exploratory attempt produces visible, meaningful progress (Bahroun et al., 2023). This transformation of abstract creativity into observable and accumulable indicators of competence enables learners to tangibly perceive their own growth.

There is an interaction between interest and cognitive competence (Fryer and Ainley, 2019). Students who combine high personal interest with strong mastery goals report higher levels of cognitive ability, indicating that interest can predict self-assessments of competence across different activities (Roure and Lentillon-Kaestner, 2022). Interest not only stimulates short-term engagement but also helps students develop a stable learner identity—when learners perceive themselves as engaged participants with genuine interest, they are more likely to consider themselves competent in the respective domain (McPhail et al., 2000). The pattern linking interest to perceived competence is observed across diverse national contexts, although the strength of the association may vary due to cultural norms and differences in educational systems (Henderson et al., 1999). Tailoring tasks to students’ interests has been shown to enhance cognitive competence, suggesting that designs aligned with learners’ interests can directly improve their self-assessments of ability (Roure and Pasco, 2022).

Over time, this reciprocal relationship between creative interest and perceived competence evolves into a self-reinforcing cycle: heightened interest promotes deeper engagement and skill acquisition, which in turn boosts learners’ confidence and further sustains their creative motivation.

*Hypothesis 7 (H7)*: Creative interest has a significantly positive effect on perceived creative competence.

### 2.5 Creative self-efficacy and perceived creative competence

Creative self-efficacy refers to an individual’s belief in their ability to generate novel and useful ideas (Tierney and Farmer, 2011). Research has shown that learners with high creative self-efficacy are more likely to perceive themselves as competent problem-solvers and demonstrate stronger intrinsic motivation for autonomous exploration throughout the creative process (Tierney and Farmer, 2002).

When such learners engage in collaboration with GAI, the mechanisms that drive the development of self-efficacy exhibit unique advantages (Preciado and Vallejo, 2025). GAI offers immediate creative outputs that serve as valuable reference points for learners (Marzano, 2025). By selecting, recombining, or critically refining these AI-generated suggestions, learners accumulate mastery experiences that contribute directly to the enhancement of their creative competence.

A substantial body of research in educational contexts has demonstrated a positive association between self-efficacy and perceived competence. For instance, academic self-efficacy has been shown to enhance intrinsic motivation and improve learners’ perceived competence across a variety of learning tasks (Buch et al., 2015). Similarly, higher general self-efficacy is associated with stronger perceived competence and greater adaptability, suggesting that self-beliefs constitute a central psychological foundation for competence development (Orkaizagirre-Gómara et al., 2020). In the domain of writing, confidence has been identified as a key determinant of perceived competence, self-efficacy, and ultimate performance outcomes (Pajares and Johnson, 1994). Self-efficacy plays a key role in shaping perceived competence across different learning settings, providing a foundation for its positive impact in creative educational environments.

*Hypothesis 8 (H8)*: Creative self-efficacy has a significantly positive effect on perceived creative competence.

### 2.6 Self-regulated learning and perceived creative competence

Self-regulated learning (SRL) primarily encompasses three key dimensions: metacognitive strategies, goal-oriented monitoring, and emotional regulation (Puustinen and Pulkkinen, 2001). In the context of GAI-supported collaborative music creation, SRL plays a critical role in determining how effectively students engage with and benefit from advanced technological tools.

Learners with strong SRL capabilities are more likely to set clear creative goals, select appropriate strategies, and continuously monitor their progress toward achieving expected creative outcomes (DiFrancesca et al., 2016). This metacognitive engagement enables them to leverage their creative interest and self-efficacy beliefs more fully, transforming psychological motivation into tangible improvements in perceived creative competence.

Research has shown that SRL can significantly predict learners’ perceived competence in complex learning environments (Pelikan et al., 2021). Similarly, the role of SRL in enhancing communicative competence has been emphasized, indicating that learners who can regulate their speaking motivation exhibit stronger perceived speaking ability (Uztosun, 2021). A substantial body of research further demonstrates that SRL has a positive effect on students’ perceived competence. By enabling learners to monitor, evaluate, and refine their strategies, SRL enhances their perception of both writing ability and actual writing performance (Bai et al., 2024; Bai and Wang, 2023). SRL also moderates the impact of executive functions on competence development (Grüneisen et al., 2023). Moreover, SRL helps students manage stress and respond more effectively to challenges, thereby facilitating improvements in perceived competence (Dörrenbächer-Ulrich et al., 2024).

Therefore, SRL functions as a key moderating factor shaping how learners translate affective-motivational states—such as creative interest and self-efficacy—into actual creative outcomes. Its presence or absence can significantly influence the depth and effectiveness of learners’ interactions with GAI, ultimately impacting their perceived creative growth (Huang et al., 2025).

*Hypothesis 9 (H9)*: Self-regulated learning has a significantly positive effect on perceived creative competence.

## 3 Methods

### 3.1 Participants

Using a convenience sampling method, a teaching experiment was conducted at a university with a racially homogeneous sample. Participants were music majors who had completed courses in music composition. Within the university, classes offering music composition were randomly selected and assigned to either the experimental group or the control group. Students were required to complete creative tasks either supported by GAI or without such support. Prior to the experiment, participants were randomly assigned to the control condition (no GAI use) or the experimental condition (using GAI). They were informed that participation was voluntary and anonymous, with the option to withdraw at any time without providing reasons. Confidentiality of their data was strictly maintained. Subsequently, an additional experiment was conducted to facilitate student collaboration in music creation using GAI, aiming to explore the pathways influencing students’ perceptions of their creative abilities. The study involved 405 participants aged between 17 and 23 years. Females constituted the majority, with 238 individuals representing 58% of the total sample. Ethical approval was obtained from the institutional ethics committee affiliated with the first author, in accordance with APA ethical guidelines.

### 3.2 Experimental procedure

The research spanned 13 weeks, with four class hours per week. During the first and second weeks, the instructor introduced the course syllabus, fundamental technical operations, and core concepts such as creativity, providing students with a comprehensive understanding of the course framework. From the third to the eighth week, students engaged in creative design activities under guided supervision. Upon completion, students were required to submit their works and complete questionnaires assessing creative self-efficacy and creativity. The specific teaching arrangement is as follows:

1. Preparation Phase (Week 1). Offline training was conducted to familiarize students with the use of AI tools and introduce relevant teaching methods. Students in the experimental group were guided to perform hands-on operations involving AI tool interventions, allowing them to experience AI-generated lyrics, melodies, and other musical elements, and to attempt personalized modifications. Specifically, the Wenxin Yi Yan tool and the AI music generation platform^1^ were utilized, enabling students to integrate their own experience and inspiration through engagement with AI-generated music.
2. Implementation Phase (Weeks 2 to 12). The course is divided into two modules. Module 1 (Weeks 2–8) covers the course outline and introduces fundamental music theory concepts such as rhythm and tempo, accompanied by relevant teaching materials for learners. Module 2 (Weeks 9–12) involves assigning creative tasks where students are required to submit a complete musical composition, including lyrics and music. The experimental group utilizes AI tools to assist in lyric writing and music composition, whereas the control group completes these tasks independently without AI assistance.
3. Evaluation phase (Week 13). Students submit their works and complete the questionnaires. Simultaneously, professional instructors conduct thorough reviews and validations of the submissions.

### 3.3 Measures

This study employed a structured questionnaire comprising five key constructs: Generative AI Usage, Self-Regulated Learning, Creative Interest, Creative Self-Efficacy, and Perceived Creative Competence. All items were measured using a 5-point Likert scale ranging from 1 (strongly disagree) to 5 (strongly agree).

#### 3.3.1 Generative AI usage

Generative AI usage was measured using four items developed based on recent literature and observed student behaviors in creative tasks. These items assessed the degree to which students integrated generative AI tools into their learning and artistic practices. For example, items included statements such as “I use generative AI tools to help me search for information” and “I try to integrate and recreate outputs from different generative AI tools.” The measurement captured both the instrumental functions of AI, such as supporting draft development, and its creative applications, including the synthesis of multimodal outputs.

#### 3.3.2 Self-regulated learning

Self-regulated learning (SRL) was assessed through three sub-dimensions: Metacognitive Strategies, Goal-Oriented Monitoring, and Emotional Control. Metacognitive Strategies were measured using three items adapted from established SRL scales (Teng and Zhang, 2016), such as “When learning a new skill or knowledge, I try to find the best way to learn it.” Goal-Oriented Monitoring comprised six items specifically tailored to the creative writing context, capturing learners’ ability to set goals, monitor progress, and evaluate performance during writing tasks (e.g., “I evaluate how well I have mastered the knowledge and skills taught in writing classes”). Emotional Control was measured by three items (e.g., “When I feel like giving up, I motivate myself to continue writing”), assessing students’ capacity to manage anxiety and sustain motivation in the face of creative challenges.

#### 3.3.3 Creative interest

Creative interest was measured using three items that capture students’ intrinsic curiosity and emotional engagement in creative activities (Lunke and Meier, 2016). These items were adapted from established creativity and flow scales, including statements such as “I am very interested in participating in creative activities” and “When immersed in creative work, I often lose track of time.”

#### 3.3.4 Creative self-efficacy

Creative self-efficacy was measured using six items adapted from Tierney and Farmer’s (2002) scale, as further refined by Karwowski (2012). These items assessed students’ confidence in their capacity to creatively solve problems, generate novel ideas, and sustain creativity under challenging conditions. Sample items include: “I am confident in my creative abilities” and “I am skilled at proposing original solutions to problems.”

#### 3.3.5 Perceived creative competence

Creative identity was measured using five items adapted from Karwowski et al. (2013), designed to assess the extent to which creativity is integrated into a student’s self-concept. Sample items include: “I consider myself a creative person” and “Originality is a personal trait I value highly.”

### 3.4 Data analysis

Data were analyzed using SPSS 26.0 and AMOS 29.0. Initially, descriptive statistics and independent samples *t*-tests were performed to examine the basic characteristics and group differences. Subsequently, SEM was employed to investigate the relationships among Generative AI Usage, Self-Regulated Learning, Creative Interest, Creative Self-Efficacy, and Perceived Creative Competence. Model fit was assessed using indices including the Comparative Fit Index (CFI), Tucker–Lewis Index (TLI), Root Mean Square Error of Approximation (RMSEA), and Standardized Root Mean Square Residual (SRMR). Following Schreiber et al. (2006), a model was considered acceptable if CFI and TLI values were equal to or greater than 0.90, and RMSEA and SRMR values were equal to or less than 0.08.

## 4 Results

### 4.1 GAI enhances creative interest, creative self-efficacy and perceived creative competence

Table 1 presents the descriptive statistics and results of independent samples *t*-tests comparing the control and experimental groups. The experimental group significantly outperformed the control group across all creativity-related measures (*p* < 0.001). Specifically, creative interest was higher in the experimental group (*M* = 4.29, *SD* = 0.41) than in the control group (*M* = 2.79, *SD* = 0.44). Similarly, creative self-efficacy was greater in the experimental group (*M* = 4.24, *SD* = 0.37) compared to the control group (*M* = 2.62, *SD* = 0.30). Perceived creative competence also showed a significant advantage for the experimental group (*M* = 4.30, *SD* = 0.36) over the control group (*M* = 3.20, *SD* = 0.40). These findings provide empirical support for Hypotheses 2, 4, and 6.

**Table 1:** Descriptive statistics and comparisons between the control group and the experimental group.

Construct	Control		Experimental		*p*
	*M*	SD	*M*	SD	
Creative interest	2.79	0.44	4.29	0.41	0.000***
Creative self-efficacy	2.62	0.30	4.24	0.37	0.000***
Perceived creative competence	3.20	0.40	4.30	0.36	0.000***

***p < 0.001.

### 4.2 Measurement model analysis

Factor loadings represent the strength of the relationship between observed variables and their corresponding latent constructs. Table 2 presents the standardized factor loadings for each measurement item on its respective latent variable. Within the GAI Support construct, all four items (GS1 to GS4) exhibited high loadings ranging from 0.791 to 0.826, indicating strong internal consistency of this scale. For the Self-Regulated Learning construct, the three items (SR1 to SR3) showed robust loadings, with SR1 reaching 0.894 and SR2 and SR3 both exceeding 0.70, thus meeting accepted psychometric criteria. The Creative Interest construct’s three items had loadings between 0.679 and 0.749, suggesting acceptable convergent validity. The Creative Self-Efficacy construct, comprising six items, demonstrated consistently high loadings ranging from 0.725 to 0.798, confirming the clarity of this dimension’s structure. In the Perceived Creative Competence construct, PCC1 showed the highest loading at 0.842, while the remaining four items ranged from 0.722 to 0.755, also supporting strong convergent validity. Overall, all factor loadings were statistically significant and met recommended thresholds, thereby substantiating the convergent validity of each scale and indicating a well-fitting measurement model (Brown and Moore, 2012).

**Table 2:** Factor loadings.

Construct	Items	Factor loadings
GAI support	GS1	0.793
	GS2	0.826
	GS3	0.791
	GS4	0.794
Self-regulated	SR1	0.894
	SR2	0.726
	SR3	0.727
Creative interest	CI1	0.749
	CI2	0.714
	CI3	0.679
Creative self-efficacy	CSE1	0.729
	CSE2	0.725
	CSE3	0.798
	CSE4	0.747
	CSE5	0.792
	CSE6	0.754
Perceived creative competence	PCC1	0.842
	PCC2	0.754
	PCC3	0.755
	PCC4	0.739
	PCC5	0.722

### 4.3 Convergent validity

Table 3 presents the results of convergent validity, offering critical insights into the reliability and accuracy of the latent constructs within the measurement model. The assessment utilized average variance extracted (AVE), composite reliability (CR), and Cronbach’s alpha coefficients to evaluate indicator reliability. Cronbach’s alpha values for all constructs exceeded the widely accepted threshold of 0.70, indicating satisfactory internal consistency (Tavakol and Dennick, 2011). Similarly, CR values ranged from 0.82 to 0.89, further supporting the reliability of the latent constructs. AVE values, ranging from 0.51 to 0.64, demonstrate that each construct accounts for a substantial proportion of the variance in its associated indicators, confirming adequate convergent validity.

**Table 3:** Constructs results of Cronbach’s alpha, AVE, and CR.

Construct	Cronbach’s alpha	CR	AVE
GAI support	0.709	0.87	0.64
Self-regulated	0.795	0.82	0.61
Creative interest	0.753	0.75	0.51
Creative self-efficacy	0.765	0.89	0.57
Perceived creative competence	0.772	0.87	0.58

### 4.4 Discriminant validity

Table 4 displays the results for all indicators. Following the criteria proposed by Fornell and Larcker (1981), the square roots of the AVE for each construct should exceed the correlations between that construct and all other constructs, thereby supporting discriminant validity.

**Table 4:** Discriminant validity.

Construct	GAI support	Creative self efficacy	Creative interest	Self-regulated	Perceived creative competence
GAI support	0.800				
Creative self efficacy	0.557***	0.754			
Creative interest	0.616***	0.343***	7.714		
Self-regulated	0.473***	0.263***	0.292***	0.781	
Perceived creative competence	0.772***	0.706***	0.585***	0.501***	0.761
AVE	0.64	0.57	0.51	0.61	0.58

***p < 0.001.

### 4.5 Structural model

**Figure 1:**
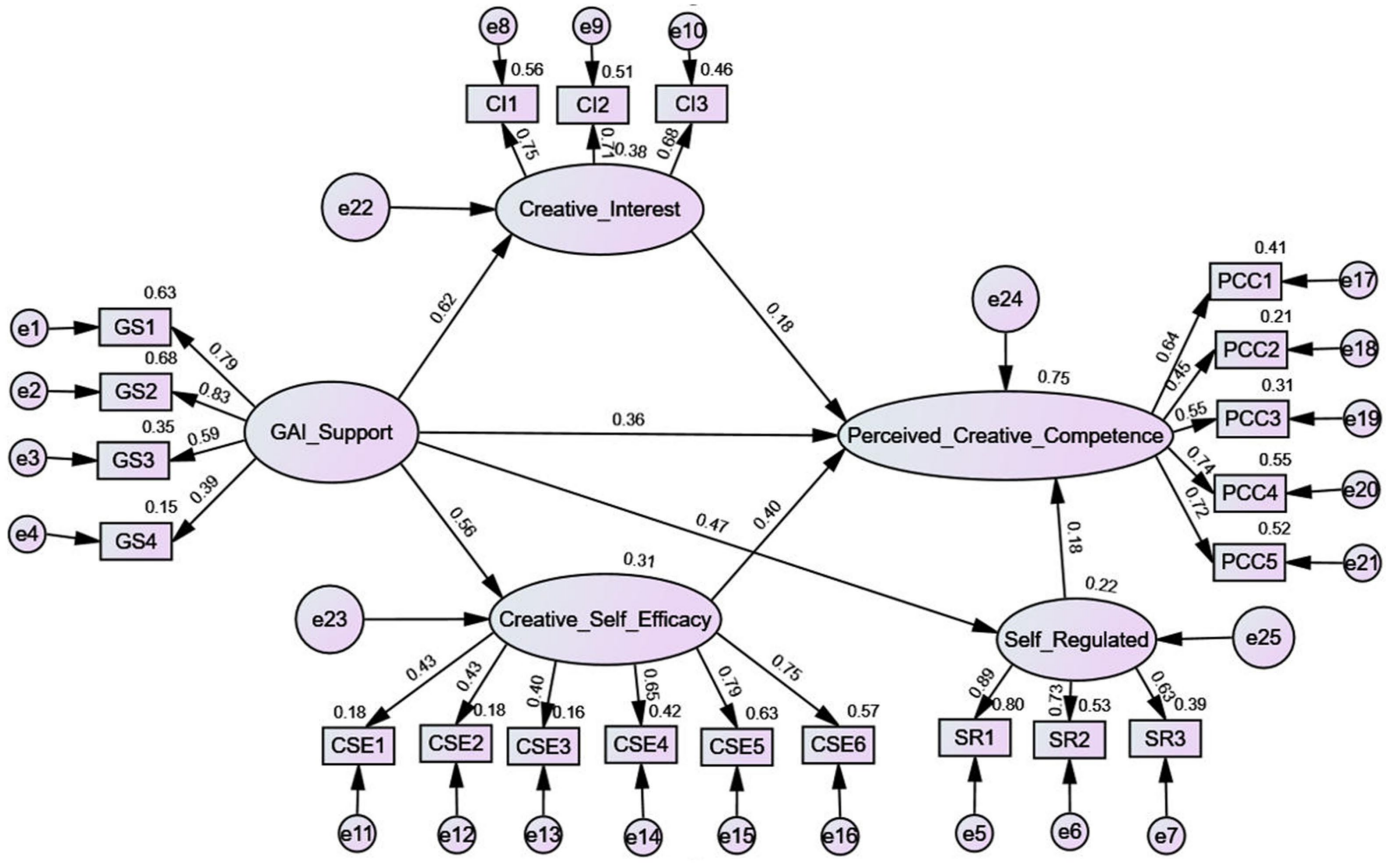
Model path.

The model fit indices indicated a satisfactory fit: *χ*^2^/df = 2.3, GFI = 0.96, AGFI = 0.93, SRMR = 0.03, RMSEA = 0.04, TLI = 0.97, and CFI = 0.98. These results demonstrate that the structural model adequately fits the data. The SEM results revealed that all hypothesized relationships were statistically significant at the *p* < 0.001 level. Specifically, GAI support significantly predicted creative interest (*β* = 0.616, *SE* = 0.063, *p* < 0.001), creative self-efficacy (*β* = 0.557, *SE* = 0.052, *p* < 0.001), self-regulated learning (*β* = 0.473, *SE* = 0.083, *p* < 0.001), and perceived creative competence (*β* = 0.357, *SE* = 0.066, *p* < 0.001). Additionally, creative interest (*β* = 0.176, *SE* = 0.051, *p* < 0.001), creative self-efficacy (*β* = 0.401, *SE* = 0.093, *p* < 0.001), and self-regulated learning (*β* = 0.175, *SE* = 0.027, *p* < 0.001) each had significant positive effects on perceived creative competence. Overall, the findings confirm the validity of Hypotheses 1, 3, 5, 7, 8, and 9, as presented in Figure 1 and Table 5.

**Table 5:** Summary of hypotheses testing results.

Regressed variables	Standardized estimate	SE	*P*	Decision
Creative interest ← GAI support	0.616	0.063	0.000***	Accept
Creative self-efficacy ← GAI support	0.557	0.052	0.000***	Accept
Self-regulated ← GAI support	0.473	0.083	0.000***	Accept
Perceived creative competence ← GAI support	0.357	0.066	0.000***	Accept
Perceived creative competence ← Creative interest	0.176	0.051	0.000***	Accept
Perceived creative competence ← Creative self-efficacy	0.401	0.093	0.000***	Accept
Perceived creative competence ←Self-regulated	0.175	0.027	0.000***	Accept

***p < 0.001.

## 5 Discussion

This study provides robust evidence for the role of GAI in facilitating the development of musical creativity within collaborative learning environments. Comparative analysis of the experimental and control groups across three key variables—creative interest, creative self-efficacy, and perceived creative competence—revealed significant differences favoring the experimental group. These findings validate the positive impact of GAI on students’ musical creativity development. Furthermore, SEM results demonstrated that GAI support significantly enhances students’ creative interest, creative self-efficacy, self-regulated learning, and perceived creative competence. This suggests that GAI functions not only as a technological tool in educational settings but also as a multifaceted support system that integrates mechanisms for motivational activation, self-efficacy reconstruction, and cognitive regulation (Kumar et al., 2023).

Among all the examined path relationships, GAI exerted the strongest influence on creative interest (*β* = 0.616), indicating that the use of GAI tools during the creative process significantly stimulates students’ curiosity. Moreover, students in the experimental group demonstrated significantly higher levels of creative interest compared to the control group, suggesting that GAI-supported collaborative creation effectively fosters intrinsic curiosity. This aligns with prior research indicating that external environmental factors characterized by support, interactivity, and timely feedback can ignite situational interest, which may subsequently internalize into enduring individual interest (Chiasson et al., 2024). By continuously responding to student inputs, offering real-time feedback, and encouraging exploration, GAI creates an intelligent and immersive creative environment that helps prevent declines in interest when students encounter challenges in the creative process.

The support provided by GAI for enhancing the development of creative self-efficacy was also highly significant (*β* = 0.557), consistent with prior findings that GAI improves students’ writing self-efficacy (Pellas, 2023). Furthermore, students in the experimental group exhibited significantly higher levels of creative self-efficacy than those in the control group, further validating that the generative mechanisms of GAI—through immediate feedback and diverse creative pathways—strengthen students’ belief in their creative abilities. During AI-supported creative activities, students gain mastery experiences by collaboratively completing complex tasks, such as composing melodies and integrating lyrics, with the system. The positive feedback and generation suggestions provided by GAI reinforce these successful experiences and enhance intrinsic motivation (Monzon and Hays, 2025).

Additionally, the study revealed that GAI significantly improved students’ self-regulated learning ability (*β* = 0.473). From a metacognitive perspective, this finding supports the positive relationship between technology-enabled regulation and learner capacity. Under AI intervention, students continuously adjust their goals, verify the logic of inputs and outputs, and regulate their emotional states. This aligns with Xu et al. (2025), who emphasize that GAI functions not as a passive tool but as an active agent that stimulates students’ cognitive loops of planning, monitoring, and adjustment.

Moreover, students’ perceived creative competence was directly influenced by GAI support (*β* = 0.357) and was also significantly predicted by creative interest (*β* = 0.176), creative self-efficacy (*β* = 0.401), and self-regulation ability (*β* = 0.175). The experimental group scored significantly higher on perceived creative competence compared to the control group, providing empirical evidence for AI-driven reconstruction of learning pathways. These findings suggest that students’ perceptions of their own creativity not only derive from individual traits but can also be shaped and enhanced through appropriate technological support.

### 5.1 Limitations and future research

Although this study highlights the positive role of GAI in supporting students’ music education and fostering their creativity, several limitations remain that warrant further investigation. First, the sample was limited to undergraduate students from a single university in China, which may constrain the generalizability of the findings. Future research should expand the sample to include learners from diverse educational stages—such as secondary school students, graduate students, and lifelong learners—to examine the universality and potential variations of GAI’s effects on creativity development across different age groups and educational backgrounds. Second, this study did not incorporate the role of teachers as a variable. In authentic educational settings, teachers’ instructional strategies, attitudes toward technology, and support behaviors often serve as crucial mediators or moderators in AI integration processes. Future studies could delve deeper into how teachers influence students’ creative motivation and learning outcomes during GAI-supported creative activities, particularly through guidance, feedback, and contextual design.

## 6 Conclusion

This study empirically confirmed the positive impact of GAI on students’ creative development through SEM. The findings confirm that GAI support significantly promotes students’ creative interest (H1), with the experimental group showing higher levels of interest compared to the control group (H2). GAI support positively influenced creative self-efficacy (H3), and students using GAI demonstrated higher self-efficacy than those in the control group (H4). Perceived creative competence was also directly enhanced by GAI support (H5), with the experimental group reporting higher competence levels than the control group (H6). Furthermore, the study highlights the mediating roles of creative interest, creative self-efficacy, and self-regulated learning in shaping perceived creative competence. Creative interest (H7) and creative self-efficacy (H8) were positively associated with perceived creative competence, illustrating the interdependent nature of motivation, belief in capability, and self-perceived creative ability. Self-regulated learning also had a significant positive effect on perceived creative competence (H9), emphasizing the importance of learners’ active monitoring, goal-setting, and strategic regulation in translating motivational and cognitive states into tangible creative outcomes. The findings indicate that GAI not only significantly enhances students’ creative interest and self-efficacy but also strengthens their self-regulated learning abilities and perceived creative competence, underscoring its broad potential in facilitating creative learning. When effectively integrated into collaborative creative tasks such as music composition, GAI increases student engagement and bolsters their creative confidence and self-perception of creative abilities. Consequently, educators and instructional designers should reconsider the role of GAI tools—not merely as auxiliary technologies, but as active co-creative partners capable of continuously fostering students’ learning motivation and long-term creative potential.

## Data Availability

The original contributions presented in the study are included in the article/supplementary material, further inquiries can be directed to the corresponding author.

## Footnotes

1
https://musichero.ai/zh-CN/app
